# Clinical Considerations Regarding Pacemaker Syndrome Under VVIR Pacing

**DOI:** 10.1002/joa3.70255

**Published:** 2025-12-08

**Authors:** Naoya Kataoka, Teruhiko Imamura

**Affiliations:** ^1^ Second Department of Internal Medicine University of Toyama Toyama Japan

**Keywords:** heart failure, pacemaker, sick sinus syndrome

To Editor,

The authors should be commended for conducting a rigorously designed study in which pacemaker syndrome (PMS) was defined using both subjective symptoms and objective improvement following restoration of AV synchrony [1]. This approach is particularly important because the clinical diagnosis of PMS is often challenging, especially in older adults whose symptoms are sometimes nonspecific. Their finding that a higher physical activity index predicts PMS is intuitively acceptable, yet it is meaningful that this relationship was demonstrated quantitatively. Several issues merit further clarification and discussion.

In this study, PMS was evaluated after switching all dual‐chamber pacemakers to VVIR mode at discharge [1]. In real‐world practice, however, VVIR pacing is rarely selected in patients with intact sinus rhythm, except in limited settings such as permanent atrial fibrillation with bradycardia. Modern leadless pacemakers now provide VDD (Micra AV) or DDD (Aveir DR) capabilities, and VVIR activation during sinus rhythm is uncommon in contemporary clinical practice [2, 3]. Therefore, we would appreciate clarification regarding the clinical scenarios the authors envision where these findings would alter pacing‐mode decision‐making. Does the discussion refer to older single‐chamber VVI devices, resource‐limited settings, or a theoretical framework for physiological understanding? The relevance of PMS induced under intentionally dyssynchronous conditions, which are rarely encountered in modern practice, deserves further elaboration.

The authors propose that increased physical activity exacerbates atrial contraction against a closed mitral valve, leading to PMS [1]. However, this explanation is based on physiologic speculation rather than direct hemodynamic measurements. An alternative mechanism may exist in patients with preserved sinus node function but impaired AV conduction. During exercise, the sinus rate may exceed the VVIR pacing rate at a certain workload, causing intermittent intrinsic beats with higher degrees of AV block. This could reproduce the hemodynamics of advanced AV block and provoke exertional dyspnea even without classical PMS mechanisms.

Cardiopulmonary exercise testing combined with pacing‐mode changes might have provided objective insight into the precise workload at which symptoms began. Such physiologic data could meaningfully strengthen the mechanistic conclusions.

The authors briefly mention dyssynchrony as a contributor to PMS [1]. Given the rapid adoption of conduction‐system pacing (His‐bundle or left bundle branch pacing), which substantially reduces ventricular dyssynchrony, it would be valuable to consider whether PMS occurs—or is mitigated—under conduction‐system pacing strategies [4]. This question is clinically relevant because newer pacing modalities aim to preserve or restore physiologic synchrony, potentially altering the incidence or expression of PMS.

## Funding

The authors have nothing to report.

## Ethics Statement

The authors have nothing to report.

## Consent

The authors have nothing to report.

## Conflicts of Interest

The authors declare no conflicts of interest.

## Data Availability

The authors have nothing to report.
